# Spatial distribution of loose connective tissues on the anterior hip joint capsule: a combination of cadaveric and in-vivo study

**DOI:** 10.1038/s41598-021-02381-1

**Published:** 2021-11-24

**Authors:** Masahiro Tsutsumi, Akimoto Nimura, Hajime Utsunomiya, Shintarou Kudo, Keiichi Akita

**Affiliations:** 1 Department of Clinical Anatomy, Graduate School of Medical and Dental Sciences, Tokyo Medical and Dental University, 1-5-45 Yushima, Bunkyo-ku, Tokyo, 113-8519 Japan; 2 Inclusive Medical Science Research Institute, Morinomiya University of Medical Sciences, Osaka, Japan; 3 Department of Functional Joint Anatomy, Graduate School of Medical and Dental Sciences, Tokyo Medical and Dental University, Tokyo, Japan; 4 Tokyo Sports & Orthopaedic Clinic, Tokyo, Japan

**Keywords:** Musculoskeletal system, Orthopaedics

## Abstract

Recently, pathological changes in the fat pad on the anterior inferior iliac spine (AIIS), between the proximal rectus femoris and joint capsule, have been highlighted as a cause of anterior hip pain. However, precise fat pad features, such as the spatial distribution distal to the AIIS, histological features, and in vivo tissue elasticity, remain unclear. This study aimed to investigate the morphological characteristics of the fat pad on the AIIS. Four hips from four cadaveric donors were both macroscopically and histologically investigated, and eight hips from four volunteers were assessed using ultrasonography. The fat pad on the AIIS was also surrounded by the iliopsoas and gluteus minimus, extending distally to the superficial portion of the vastus lateralis, and the anterior portion of the gluteus maximus tendon. Histological analysis revealed that the fat pad was composed of loose connective tissue. Based on the ultrasonography, the shear wave velocity in the fat pad was significantly lower than that in the joint capsule. Conclusively, the pathological adhesion between the joint capsule and pericapsular muscles, if caused by fat pad fibrosis, may occur following the abovementioned fat pad spatial distribution.

## Introduction

Intra-articular hip pathologies, such as labral tear and cartilage damage, are considered as major causes of hip pain. However, extra-articular hip pathologies (such as pericapsular tendinosis and postoperative pericapsular muscle and joint capsule adhesions) have recently received attention since interventions for these pathologies (surgery or ultrasound-guided injection) were effective^1,2^. Loose connective tissue generally exists around pericapsular muscle tendons or in the interspace between pericapsular muscles and joint capsules; this connective tissue contributes in promoting muscle force transmission and dissipating stress on the muscle attachment site^3,4^. Therefore, understanding the morphological features of this loose connective tissue can facilitate the comprehension of extra-articular hip pathology; nonetheless, there is a limitation in the basic knowledge of the morphological features of the loose connective tissue.

According to recent studies, pathological changes in the fat pad located on the anterior inferior iliac spine (AIIS)—between the proximal rectus femoris and hip joint capsule—are closely related to anterior hip pain development^1,5^. Although the rectus femoris, iliopsoas and hip joint capsule, which is identical to the iliofemoral ligament, attach onto the AIIS^6–9^, few studies have evaluated the distal spread of the fat pad on the AIIS, especially the positional relationship of the fat pad with the abovementioned pericapsular muscles. Moreover, the precise characteristics of the fat pad—histological features and in vivo tissue elasticity, as assessed by shear wave elastography during ultrasound imaging^10^—remain unclear. Both the spatial distribution and the abovementioned characteristics of the fat pad on the AIIS may clarify the pathological changes in the fat pad that play a role in anterior hip pain development.

This study aimed to investigate the morphological features of the fat pad on the AIIS, focusing on its spatial distribution distal to the AIIS, histological features, and in vivo tissue elasticity. We hypothesized that the fat pad on the AIIS spreads distal to the joint capsule, is composed of loose connective tissue, and is significantly less elastic than the joint capsule, which is a dense connective tissue.

## Methods

### Cadaveric specimen preparation

Four hips from four Japanese cadavers (two males and two females; mean age at death 73.8 years) that were donated to the Department of Anatomy, Tokyo Medical and Dental University, were used in this study. All donors voluntarily declared before their death that their remains would be donated for education and study, and our study complies with the Japanese law entitled, “Act on Body Donation for Medical and Dental Education”. The study design was approved by the Medical Research Ethics Committee of Tokyo Medical and Dental University (#M2018-044), and all procedures were performed in accordance with the Japanese guideline entitled, “Ethical Guidelines for Medical and Health Research Involving Human Subjects”. We included cadavers that had no apparent osteoarthritic changes and did not undergo hip surgery during their lifetime. Two persons (M. T. and A. N.) specializing in anatomy, with a Doctor of Philosophy degree in Medical Science, independently examined the cadaveric specimens.

All cadaveric specimens were fixed with 8% formalin and preserved in 30% ethanol. The skin and subcutaneous tissues were removed to enable the dissection of the anterior region of the hip joint capsule, while preserving the aponeurosis and fascia superficial to the gluteal and thigh muscles. By removing the medial two-thirds of the gluteus maximus, the proximal osseous shape of the femur was identified, and the parts of all specimens—posteromedial to the iliopsoas and intertrochanteric crest—were cut and removed using a diamond saw (EXAKT 312; EXAKT Advanced Technologies, Norderstedt, Germany). In addition, parts of the specimens, proximal to the AIIS and distal to the gluteal tuberosity, were cut and removed using the diamond saw. In order to determine if any bony or soft tissue abnormalities were present, the sectioned specimens underwent micro-computed tomography (micro-CT) (inspeXio smx-100ct; Shimazdu, Kyoto, Japan) with a 200-µm resolution. Three-dimensional micro-CT images were reconstructed using an application software (ImageJ version 1.53; National Institutes of Health, Bethesda, MD, USA), and no obvious bony abnormalities were observed in all specimens. Therefore, all four specimens were subjected to both macroscopic and histological analyses.

### Macroscopic analysis: Serial axial sections of the anterior region of the joint capsule

All four specimens were embedded in a 3% agar solution and frozen at − 80 °C. The specimens were serially sectioned into 5-mm thick segments, parallel to the short axis of the femur, using a band saw (WN-25-3; Nakajima Seisakusho, Osaka, Japan); the specimens were sectioned from the AIIS to the gluteal tuberosity. Each axial section level was identified based on the correspondence between the axial section bony morphology and that observed on the micro-CT image. Excess agar was removed, after which each axial section was observed without dissection.

### Histological analysis of the axial sections of the anterior region of the joint capsule

**Figure 1:**
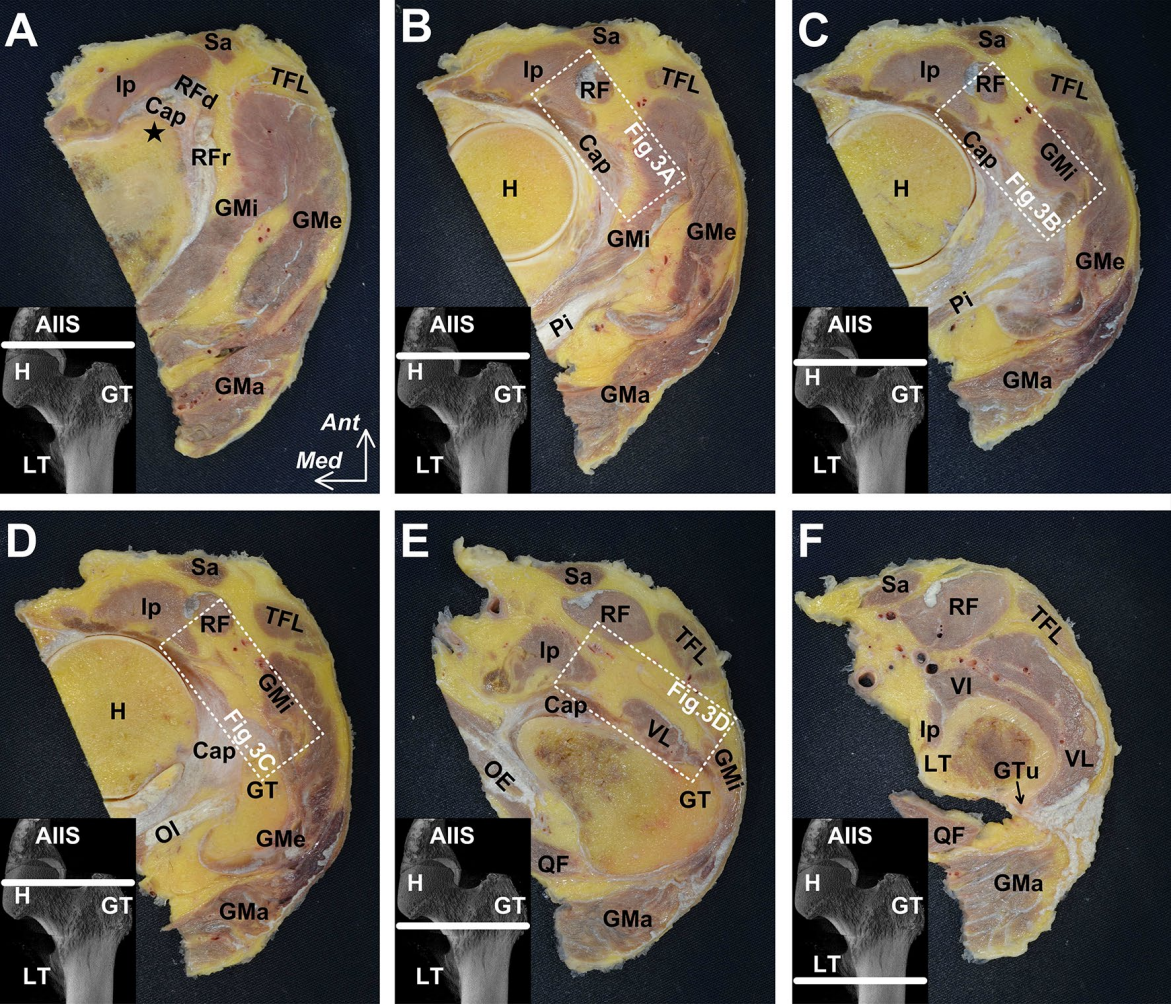
Serial axial sections of the anterior region of the joint capsule. The locations of each axial section are indicated using white lines, in each lower left panel, on the micro-CT images of the anterior aspect of the left hip (**A**–**F**). The most proximal level (**A**) is the inferior area of the anterior inferior iliac spine (indicated by the star), and the most distal level (**F**) is the superior end of the gluteal tuberosity (GTu). The boxed regions indicate the selected parts for histological analysis, as shown in Fig. 3. The fat pad superficial to the joint capsule (Cap) spreads distally to the superficial portion of the vastus lateralis (VL). *AIIS* anterior inferior iliac spine, *GMa* gluteus maximus, *GMe* gluteus medius, *GMi* gluteus minimus, *GT* greater trochanter, *H* head of femur, *Ip* iliopsoas, *LT* lesser trochanter, *OE* obturator externus, *OI* obturator internus, *Pi* piriformis, *QF* quadratus femoris, *RF* rectus femoris, *RFd* direct head of the RF, *RFr* reflected head of the RF, *Sa* sartorius, *TFL* tensor fasciae latae, *VI* vastus intermedius, *VL* vastus lateralis; *Ant* anterior, *Med* medial.

After macroscopic analysis, we chose axial sections at the following four levels: immediately inferior to the anterosuperior acetabular margin, superior to the greater trochanter, the superolateral end of the intertrochanteric line, and the mid-intertrochanteric line (Fig. 1; the boxed regions indicate the selected parts for histological analysis). Subsequently, we harvested a block from each axial section, including the region between the rectus femoris and joint capsule; the blocks were decalcified for 2 weeks with Plank-Rychlo solution (AlCl_3_:6H_2_O [70.0 g/L], HCl [85.0 mL/L], and HCOOH [50.0 mL/L])^11^. After decalcification, each block was dehydrated and embedded in paraffin. The paraffin-embedded tissue was sliced into 5-μm sections with 1-mm interval. There were approximately four sections per one block (mean proportion of the fat pad, 56.4%); the sections were stained using the Masson trichrome staining protocol.

### In vivo axial ultrasound assessment of the anterior region of the joint capsule

Ten hips of five healthy volunteers (five males; mean age, 34.2 years) were investigated. Female were excluded because there are gender differences in the stiffness of connective tissues such as ligaments and tendons in many joints and the menstrual cycle effects on joint laxity in women is still being debated^12–15^.We recruited individuals with no history of previous hip surgery. The study design was also approved by the Medical Research Ethics Committee of Tokyo Medical and Dental University (#M2020-218), and all procedures were performed in accordance with the Declaration of Helsinki (last modified in 2013) and the Japanese guideline entitled, “Ethical Guidelines for Medical and Health Research Involving Human Subjects”. All potential participants were informed of the study requirements, benefits, and risks, after which those desiring to participate in the study provided written informed consent.

Ultrasound assessment of the anterior region of the joint capsule was performed, with participants lying supine in the neutral hip position, using an ultrasound scanner (Logiq E10; GE Health Care, Milwaukee, WI, USA) with a 5–18 MHz linear transducer. A short-axis B-mode image of the anterior region of the joint capsule was visualized at the level immediately inferior to the anterosuperior acetabular margin. Subsequently, a shear wave elastographic image was obtained while assessing the quality map of the image; the quality of the shear wave was presented on a color-coded map^16,17^. The transducer was kept stationary for 10–15 s, in order to record the shear wave elastographic image and assess the quality map simultaneously. We excluded two hips with a large superficial muscular volume, belonging to the same volunteer, because of poor-quality shear wave image of the joint capsule and pericapsular muscles. The large muscular volume caused the attenuation of the shear wave before its transmission to the region of interest. Therefore, eight hips of four volunteers (four males; 34.3 years) were included in the final analysis.

Based on recorded ultrasound images, the shear wave velocity of the region of interest was measured to qualitatively assess the stiffness. A region of interest with a diameter of 5 mm was set as the measurement area on the joint capsule and the fat pad deep to the rectus femoris, after confirming the presence of good-quality shear waves in the quality map of the measurement area. All measurements were performed five times by a single observer (M. T., a licensed physical therapist with a Doctor of Philosophy degree in Medical Science), and the average of five measurements was recorded for statistical analysis. Intraclass correlation coefficients (ICCs) were calculated to determine the intra-rater reliability of each measured value.

### Statistical analyses

Statistical analyses were performed using SPSS (version 27.0; IBM Corp, Armonk, NY, USA). Statistical comparisons of the shear wave velocities in two regions, the joint capsule and the fat pad deep to the rectus femoris, were performed using the Wilcoxon signed-rank test, and the significance level was set at p < 0.05. Data are presented as median and interquartile range (IQR). In addition, using the G *Power Software (version 3.1.9.6), the effect size was calculated, and post-hoc power analysis was performed. The effect size and power were 0.63 and 0.76, respectively. ICCs were also determined using a measurement process analysis of the shear wave velocities on the joint capsule and fat pad. The qualitative cut-offs for ICC values are reported as follows: poor, < 0.40; fair, 0.40–0.59; good, 0.60–0.74; and excellent, 0.75–1.0^18^. ICCs in our study participants were 0.91 [95% CI 0.74–0.98] for the joint capsule and 0.84 [95% CI 0.59–0.97] for the fat pad.

## Results

### Spatial distribution of the fat pad on the AIIS

**Figure 2:**
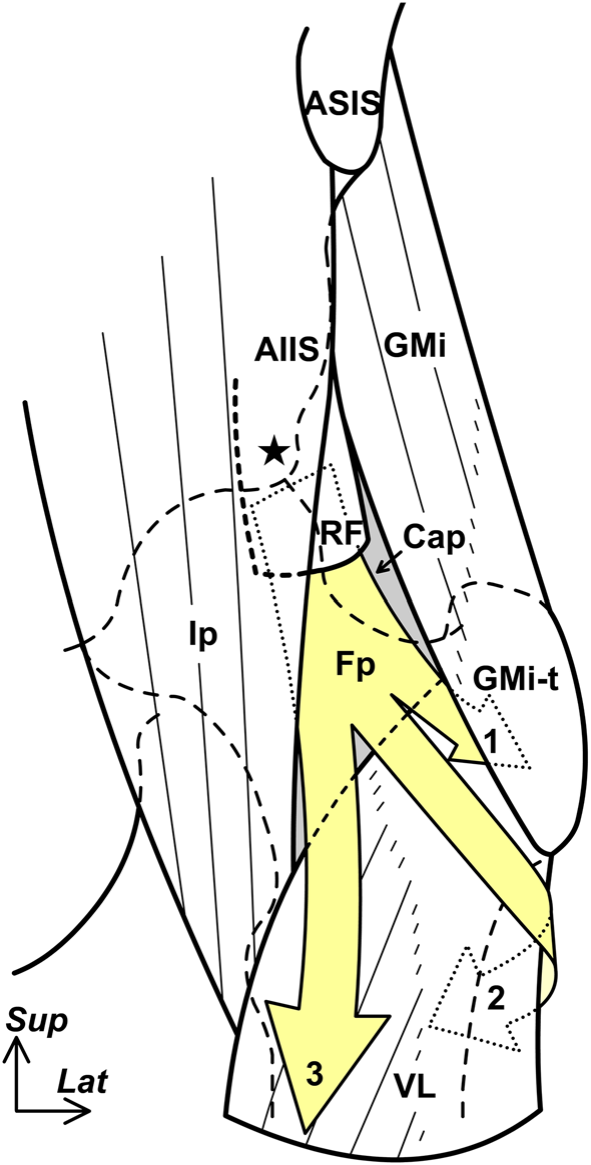
Schematic diagram indicating the spatial distribution of the fat pad on the AIIS. The schematic diagram of the anterior aspect of the left hip. The fat pad (Fp) deep to the rectus femoris (RF) and superficial to the joint capsule (Cap) were surrounded by the lateral portion of the iliopsoas (Ip) and the medial portion of the gluteus minimus (GMi). It extended distally, deep to the gluteus minimus tendon (GMi-t, fat pad extension was indicated by arrow no. 1) and posterolaterally to the anterior portion of the gluteus maximus tendon (extension was indicated by arrow no. 2) and superficial to the vastus lateralis (VL, extension was indicated by arrow no. 3). *AIIS* anterior inferior iliac spine, *ASIS* anterior superior iliac spine, Star: inferior area of the AIIS; *Lat* lateral; *Sup* superior.

Although the rectus femoris was in close contact with the joint capsule at the level of the inferior area of the AIIS (Fig. 1A), the fat pad was located deep to the rectus femoris and superficial to the joint capsule at the level immediately inferior to the anterosuperior acetabular margin (Fig. 1B). Moreover, the fat pad was surrounded by the deep lateral portion of the iliopsoas muscle and the medial portion of the gluteus minimus. At a level superior to the greater trochanter, where the gluteus minimus tendon connected to the joint capsule, the fat pad was located deep to the rectus femoris, medial to the gluteus minimus, lateral to the iliopsoas, and superficial to the joint capsule (Fig. 1C). At the superolateral end of the intertrochanteric line, the fat pad partly extended to the deep portion of the gluteus minimus tendon (Fig. 1D). Moreover, the fat pad spread distally to the superficial portion of the vastus lateralis (at the middle level of the intertrochanteric line) (Fig. 1E), and posterolaterally to the anterior portion of the lateral femoral intermuscular septum and gluteus maximus tendon (at the superior end of the gluteal tuberosity) (Fig. 1F). The abovementioned spatial distribution of the fat pad was summarized in Fig. 2.

### Histological distribution of the loose connective tissue on the anterior region of the joint capsule

**Figure 3:**
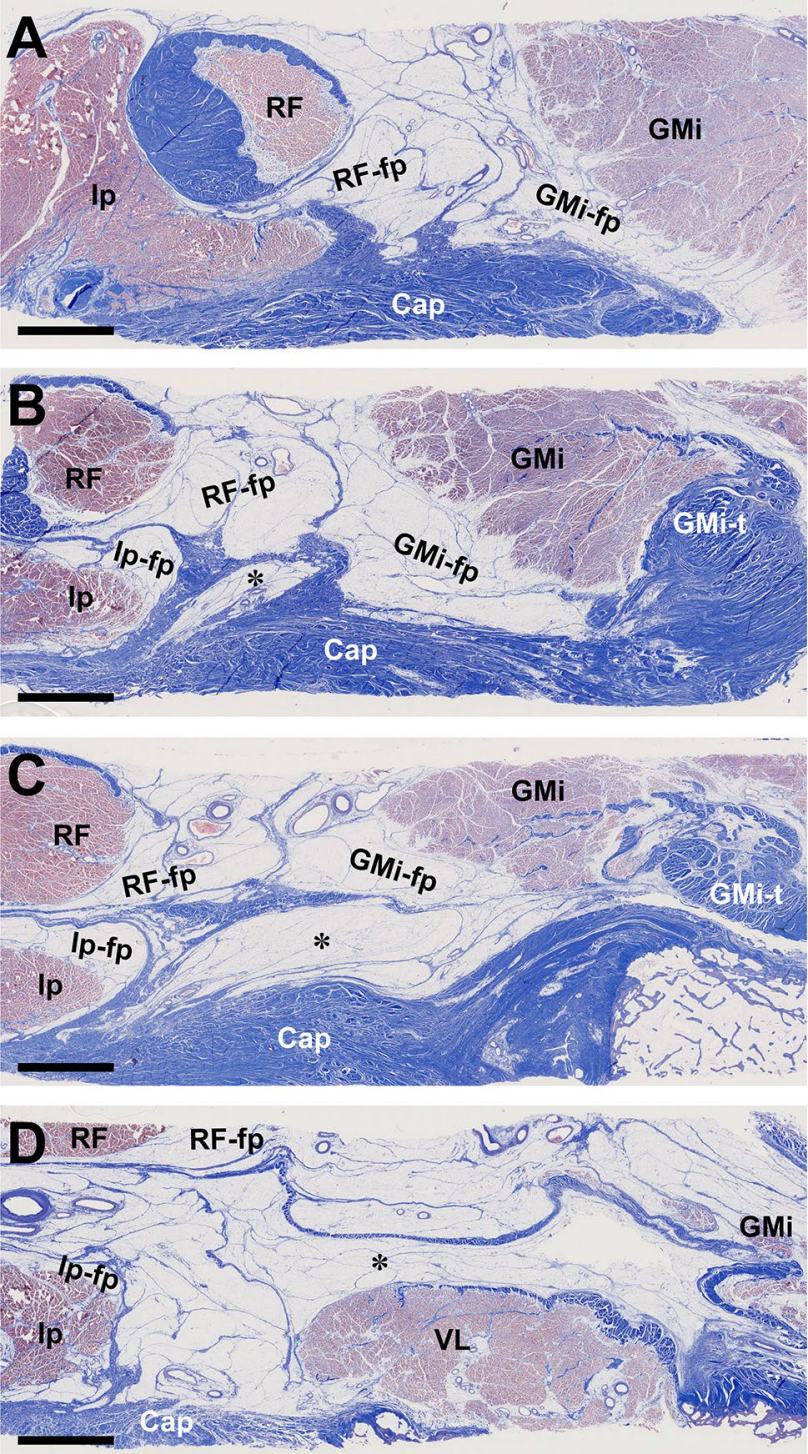
Histological distribution of loose connective tissue on the anterior region of the joint capsule (Masson's trichrome staining). Histological sections of the boxed region in Fig. 1B–E are shown in (**A**–**D**), respectively. The fat pad is segmented by a dense connective tissue and roughly divided into fat pad segments deep to the rectus femoris (RF-fp), medial to the gluteus minimus (GMi-fp), lateral to the iliopsoas (Ip-fp), and superficial to the joint capsule and vastus lateralis (indicated by asterisk). Scale bars = 5 mm. *Cap* joint capsule; *GMi* gluteus minimus, *GMi-t* GMi tendon, *Ip* iliopsoas, *RF* rectus femoris, *VL* vastus lateralis.

Histological analysis revealed that the fat pad, deep to the rectus femoris and superficial to the joint capsule, contained loose connective tissue (Fig. 3A–D). Small vessels were also distributed in the fat pad. In addition, the fat pad was segmented by dense connective tissue, and roughly divided into fat pad segments that were deep to the rectus femoris, medial to the gluteus minimus, lateral to the iliopsoas, and superficial to the joint capsule and vastus lateralis. The structure of the septum, which roughly divided the fat pad into segments, was identical to those of the deep aponeuroses of the rectus femoris, gluteus minimus, and iliopsoas, and continued with the joint capsule.

### Ultrasonographic images of the anterior region of the joint capsule

**Figure 4:**
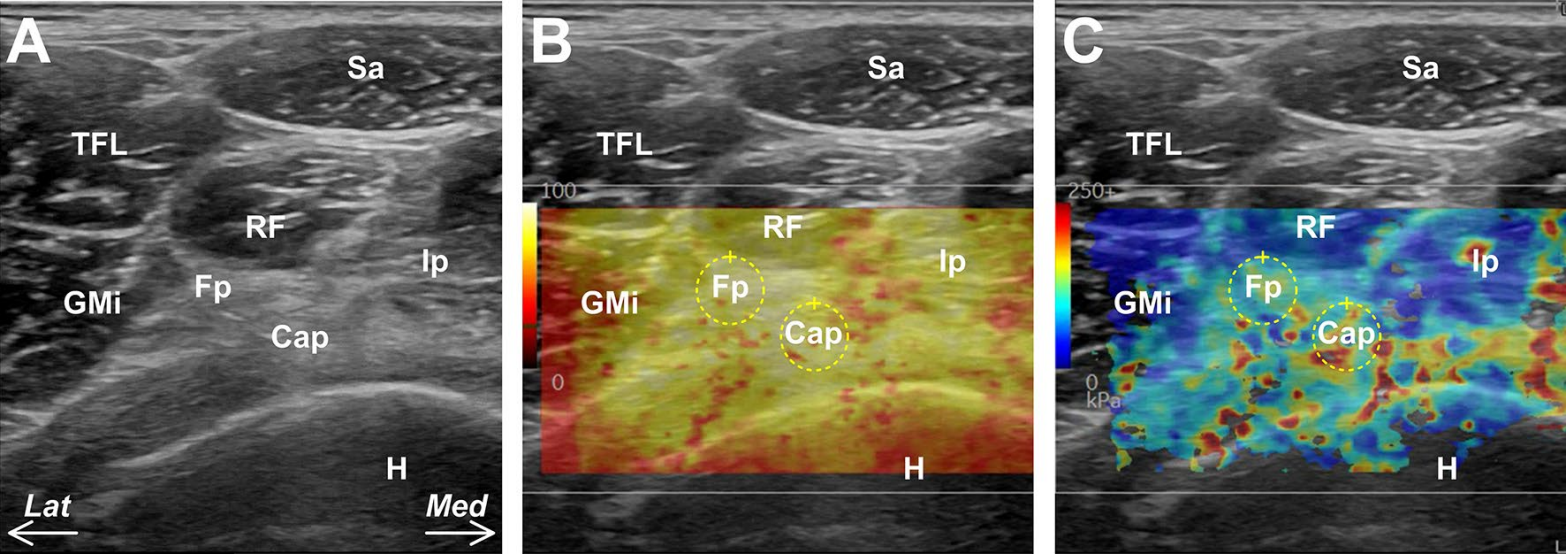
Ultrasonographic images of the anterior region of the joint capsule. (**A**) Short-axis image at the proximal level of the femur head (H). The fat pad (FP) is located deep to the rectus femoris (RF), medial to the gluteus minimus (GMi), lateral to the iliopsoas (Ip), and superficial to the joint capsule (Cap). (**B**) Quality map of the shear wave. Yellow and red represent shear waves with high and low qualities, respectively. (**C**) Shear-wave elastographic images. The red and bright colors represent high shear wave velocity, and blue and dark colors represent low shear wave velocity. *Sa* sartorius, *TFL* tensor fasciae latae; Yellow circles: measurement points of the shear wave velocity; *Lat* lateral, *Med* medial.

**Figure 5:**
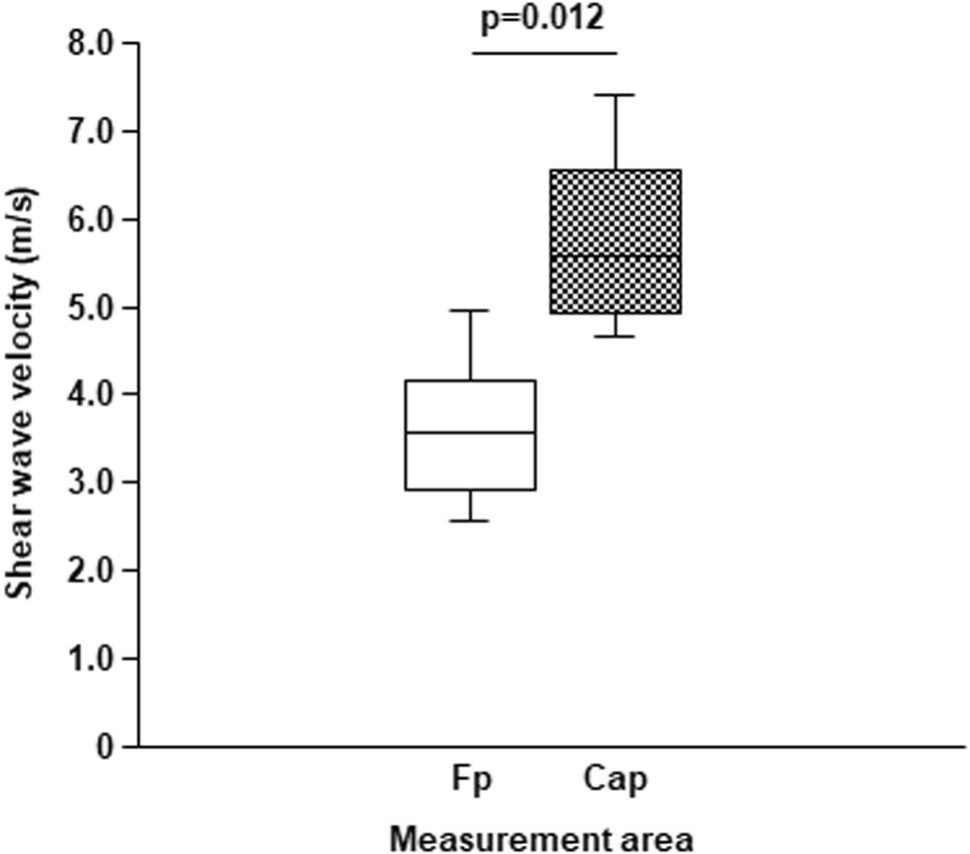
Comparison between fat pad and joint capsule shear wave velocities on the anterior region of the hip. The shear wave velocity in the fat pad (Fp) was significantly lower than that in the joint capsule (Cap).

To confirm the findings of the cadaveric analyses, we used the short-axis view of the ultrasound image to evaluate the proximal level of the femoral head, immediately inferior to the anterosuperior acetabular margin (Fig. 4A). The relatively hyperechoic region—deep to the rectus femoris, medial to the gluteus minimus, lateral to the iliopsoas and superficial to the joint capsule—was identified as the fat pad. Based on the color map on shear wave elastography, the shear wave velocities of the fat pad and joint capsule were low (median 3.5 m/s; IQR 2.7–4.2 m/s) and high (median 5.5 m/s; IQR 4.8–6.4 m/s), respectively (Fig. 4B,C). There was a significant difference between the abovementioned shear wave velocities (Fig. 5, p = 0.012).

## Discussion

The present study revealed that the fat pad between the proximal rectus femoris and the joint capsule—also surrounded by the iliopsoas and gluteus minimus—extended distally deep to the gluteus minimus tendon and superficial to the vastus lateralis, and posterolaterally to the anterior portion of the gluteus maximus tendon. The fat pad contained loose connective tissue and segmented by the deep aponeuroses of the rectus femoris, gluteus minimus, and iliopsoas. Ultrasonographic analysis revealed that the shear wave velocity in the fat pad was significantly lower than that in the joint capsule.

Regarding the spatial distribution of the fat pad on the anterior region of the hip, Walters et al. observed a fat pad between the gluteus minimus and iliocapsularis, which extended from the proximal origin of the vastus lateralis to the reflected head of the rectus femoris^19^. Although their findings were almost consistent with our findings, the superficial-deep relationships between the fat pad and pericapsular muscles, and the fat pad continuity to the anterior portion of the gluteus maximus tendon was not shown in their study. These differences might have resulted from the fact that we used serial axial sections of the anterior joint capsule region without dissections, whereas Walters et al. partly detached the pericapsular muscle and removed the fat pad itself^19^. Additionally, Kaya reported the precise positional relationships between the fat pad on the AIIS and pericapsular muscles; nonetheless, their findings were localized, and the distribution of the fat pad distal to the AIIS was not observed because they used hip arthroscopic imaging with a limited field of view^1^. Therefore, the current study might be the first to accurately and comprehensively evaluate the distribution of the fat pad on the anterior region of the hip.

Few studies have focused on the histological features of the fat pad in the anterior region of the hip. Although Kaya presented histological data^1^, they focused on the pathological changes localized on the AIIS, and did not show the relationship of the fat pad to the pericapsular muscles. Tsutsumi et al. showed the layered structures of the joint capsule at the cross sections horizontal to the intertrochanteric line^20^. Based on their histological figures, we could ascertain the presence of loose connective tissue segmented by the deep aponeuroses of the rectus femoris, gluteus minimus, and iliopsoas, although they did not mention this result in their report^20^. This loose connective tissue, which might have extended proximally to the AIIS and distally to the superficial portion of the vastus lateralis, might be identical to the fat pad in the present study. Therefore, it can be considered that the previous study supported the segmented fat pad structure revealed in the present study^20^.

As for the ultrasonographic imaging of the fat pad on the anterior region of the hip, a previous study reported the positional relationship between the fat pad and pericapsular muscles^5^; nevertheless, the previous study did not describe images with elasticity evaluation using shear wave elastography. Positional relationships among the fat pad, joint capsule, and pericapsular muscles on ultrasonography were consistent with those found in the cadavers, and the shear wave velocity in the fat pad was significantly lower than that in the joint capsule, which was considered to be due to the difference in connective tissue density between the two structures. Additionally, when we compared the fat pad stiffness among the different fat pads in the body, such as those of the knee or heel^21,22^, the fat pad stiffness in the anterior hip was relatively higher than that of the others reported regions. This difference in stiffness might be caused by dense connective tissue that segment the fat pad of the anterior hip.

Our study findings are clinically relevant. Damage to other fat pads, such as those of the knee and heel^23,24^, is considered as a contributing factor to joint pain^4^; moreover, Kaya showed that pathological changes in the fat pad on the AIIS were common in patients with anterior hip pain^1^. The present study showed that the fat pad on the AIIS extended distally deep to the gluteus minimus tendon and to the superficial portion of the vastus lateralis, and posterolaterally to the anterior portion of the gluteus maximus tendon. If the pathological adhesion between the joint capsule and pericapsular muscles, as previously reported^1^, is caused by fat pad fibrosis, such pathological adhesion might occur in accordance with the spatial distribution of the fat pad as presented in this study. Although there was no evidence to indicate that fibrosis caused pain, increased levels of connective tissue and pain might occur in tandem. It is therefore important to know the baseline values of the fat pad stiffness, like the shear wave velocity in the present study. This speculative association can be investigated further in a future study with a large sample size. Moreover, understanding the pathological change of the fat pad and its extent should be further studied to understand the development of anterior hip pain.

Our study had several limitations. First, the cadaveric and ultrasonographic evaluations were limited to uninjured specimens and healthy individuals, respectively. Therefore, the hip pathomechanism presented in this study is speculative and needs to be verified in clinical situations. Second, although we included cadavers that had no apparent osteoarthritic changes, underwent no hip surgery during their lifetime, and had no bony and soft tissue abnormalities based on the micro-CT analysis, we could not obtain the information on whether the cadaveric donor had a history of hip associated pain or lower limb pathology. Third, because we did not compare the fat pad on the AIIS with those around the body, we could not conclude how the shear wave velocities within the fat pad on the AIIS compared with those within the other fat pads around the body. Fourth, the sample size was relatively small. However, the effect size and power of the ultrasonographic data were 0.63 and 0.76, respectively, which were not low. Moreover, we compensated for the small sample size with a multidimensional analysis, including macroscopic, histological, and ultrasound analysis. Finally, the advanced age of the individuals from whom the cadaveric specimens were obtained might have affected our findings. However, the effect was expected to be minimal since the cadaveric findings were almost verified by ultrasonography.

In conclusion, the fat pad on the AIIS extended distally to the joint capsule, even to the anterior portion of the gluteus maximus tendon; was composed of loose connective tissue; and was significantly less elastic than the joint capsule. The pathological adhesion between the joint capsule and pericapsular muscles⁠—if caused by fat pad fibrosis⁠—may occur following the spatial distribution of the fat pad, as presented in this study.

## Data Availability

The datasets used and/or analyzed during the current study are available from the corresponding author upon reasonable request.
